# Role of autophagy in cadmium-induced apoptosis of primary rat osteoblasts

**DOI:** 10.1038/srep20404

**Published:** 2016-02-08

**Authors:** Wei Liu, Nannan Dai, Yi Wang, Chao Xu, Hongyan Zhao, Pengpeng Xia, Jianhong Gu, Xuezhong Liu, Jianchun Bian, Yan Yuan, Jiaqiao Zhu, Zongping Liu

**Affiliations:** 1 College of Veterinary Medicine, Yangzhou University, Yangzhou 225009, Jiangsu, China; 2 Jiangsu Co-innovation Center for Prevention and Control of Important Animal Infectious Diseases and Zoonoses, Yangzhou 225009, Jiangsu, China

## Abstract

Cadmium (Cd) is a common environmental pollutant that can damage many organs and the fetus. We previously reported that Cd induced apoptosis in primary rat osteoblasts (OBs). OB apoptosis induced by Cd will eventually lead to osteoporosis. In this study, a novel pharmacotherapeutic approach was investigated involving the regulation of autophagy to prevent Cd osteoporosis. The results showed that Cd treatment induced apoptosis in OBs, as demonstrated by the ratio of Bax/Bcl-2, activation of poly (ADP-ribose) polymerase (PARP) and nuclear condensation. In addition, cells treated with Cd were observed to undergo autophagic cell death by monitoring the induction of the beclin 1, autophagy gene 5 (Atg5) and the expression of microtubule-associated protein 1 light chain 3 (LC3). The results indicated that promotion of apoptotic cell death by Cd is accompanied by induction of autophagy in OBs. Interestingly, Cd-mediated apoptotic cell death was suppressed by pretreatment with the autophagy activator rapamycin (RAP) and potentiated by the autophagy inhibitor chloroquine (CQ) or small interfering RNA against beclin 1. These findings suggest that the autophagic response plays a protective role that impedes eventual cell death. Activation of autophagy could therefore be an adjunctive strategy for treatment of Cd-induced osteoporosis.

Cadmium is a toxic heavy metal used widely in industries that can enter the environment and stay intact for long periods of time. It can be taken up by plants, fish, and animals and enters the food chain^1^. Early study of the toxicity of Cd focused on occupational exposure, which was associated with renal dysfunction, osteomalacia, hypercalciuria and renal stone formation^2^. Recent population-based studies in Europe and China showed that a low level of cadmium exposure was associated with an increased risk of osteoporosis^3^,^4^,^5^.

Cell death, as a fundamental cellular response, plays an essential role in the development, differentiation, homeostasis and survival of organisms. Apoptotic and autophagy-based cell death are two distinct processes, which can be activated by different biochemical cascades, and display diverse morphological features. Apoptotic cell death is a highly regulated process of cell deletion and plays a fundamental role in animal development and tissue homeostasis^6^,^7^. Abnormal regulation of apoptosis increases the occurrence of human diseases, including autoimmune diseases, neurodegeneration and cancer^8^. A large number of studies have shown that Cd, as an exogenous stimulating factor, induces apoptosis in many cell types from different tissues, including liver cells^9^, immune cells^10^,^11^ and neuronal cells^12^, which can disrupt homeostasis in the adult organism. However, only a few studies^13^,^14^,^15^ have investigated apoptosis induced by Cd in osteoblasts (OBs), a cell type that plays an important role in bone remodeling. In this study, we investigated the mechanism of Cd-induced apoptosis in OBs.

Autophagy, or type II programmed cell death, is a major intracellular degradation process that delivers cytoplasmic constituents to the lysosome^16^. Recent studies demonstrated the complexity of the physiological and pathophysiological roles of autophagy, including cell death, starvation adaptation, intracellular protein and organelle clearance, development, anti-aging, tumor suppression and antigen presentation^17^. A low level of autophagy maintained in normal cells helps cells to adapt to the environment, however, excessive autophagy can trigger cell death^18^. Although many studies have demonstrated that Cd exposure induces activation of autophagy in diverse cell types^19^,^20^,^21^, few have clarified the mechanism of autophagy induced by Cd in primary rat OBs.

Apoptosis, autophagy and necrosis are different processes of cell death. An increasing number of studies have described the complex relationship between apoptosis and autophagy in different circumstances^22^, including reports that autophagy is a precondition of apoptosis^23^, autophagy inhibit apoptosis^24^,^25^, and autophagy and apoptosis promote cell death^26^,^27^. The study of apoptosis and autophagy is now focused on understanding the complex interplay between these processes for discovery of novel drugs in the treatment of cancer^28^. However, few studies have been conducted to investigate the effect of autophagy in Cd-induced apoptosis. In the current study, primary OBs were used as a model to investigate the role of autophagy in apoptosis induced by Cd, which may allow future discoveries of novel drug targets for Cd toxicity treatment.

## Results

### Cadmium induces apoptosis in osteoblasts

**Figure 1:**
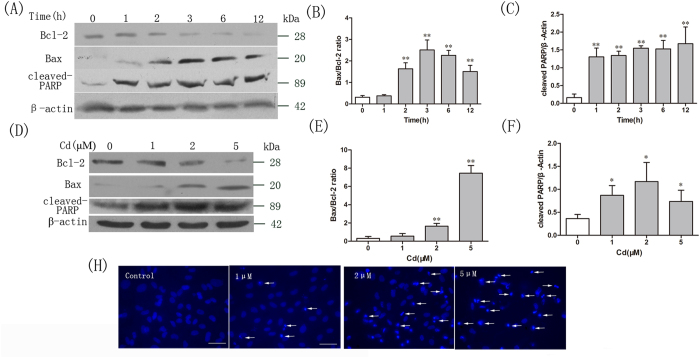
Cadmium (Cd) induces apoptosis in OBs. (**A**) Expression levels of Bax, Bcl-2 and PARP in OBs after treatment with Cd (2 μM) at different time points were detected by Western Blotting. (**D**) Western blot analysis of the Bax, Bcl-2 and cleaved-PARP in OBs treated with 1, 2 or 5 μM Cd for 3 h. Blots for Bax, Bcl-2 (BE) and cleaved PARP (CF) in OBs were semi-quantified using Image LabTM software. Data are expressed as mean ± SD (n = 3) relative to control.^*^P < 0.05 in comparison to the control by one-way ANOVA.^**^P < 0.01 in comparison to the control by one-way ANOVA. (**H**) OBs were treated with 1, 2 or 5 μM Cd for 3 h. After fixation, cells were stained with Hoechst 33258 and cell morphological characterization was analyzed using confocal fluorescence microscope. The arrows indicate apoptotic OBs showing nuclear condensation. Microphotographs were shown as representative results from three independent experiments.

To confirm that the observed cell death caused by Cd was due to apoptosis, the apoptosis parameters of OBs in response to Cd treatment were assessed by western blotting and Hoechst staining. As shown in Fig. 1A,B,D and E, changes in the expression levels of Bax and Bcl-2 proteins occurred after Cd treatment. Treatment of OBs with 2 μM Cd for various time periods caused upregulation of Bax expression in a time-dependent manner, while Bcl-2 expression was correspondingly downregulated (Fig. 1A). In addition, the poly (ADP-ribose) polymerase (PARP) protein was cleaved into its characteristic 89 kDa fragment upon treatment with Cd (Fig. 1C). OBs incubated with Cd (1, 2 and 5 μM) for 3 h showed the same regulation of expression of Bcl-2, Bax and cleaved PARP dose-dependently (Fig. 1D–F). Furthermore, nuclear morphological changes of apoptosis were observed in OBs (Fig. 1H). As indicated by strong blue fluorescence, Cd treatment caused significant increases in apoptotic cell number with condensed and fragmented DNA.

### Cd triggers autophagy in OBs

**Figure 2:**
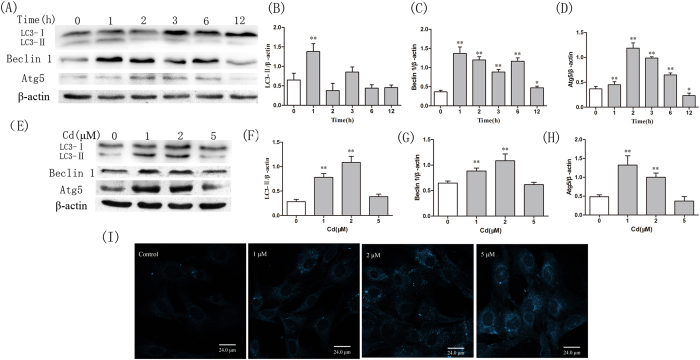
Cd induces autophagy in OBs. (**A**) LC3, beclin 1 and Atg5 expression was examined in cells treated with Cd (2 μM) at different time points. (**E**) Western blot analysis of the LC3, beclin 1 and Atg5 in cells treated with 1, 2 or 5 μM Cd for 3 h. Blots for LC3-II(BF), Beclin 1 (CG) and Atg5 (DH) in OBs were semi-quantified using Image LabTM software. Data are expressed as mean ± SD (n = 3) relative to control. ^*^P < 0.05 in comparison to the control by one-way ANOVA.^**^P < 0.01 in comparison to the control by one-way ANOVA. (**I**) OBs were incubated with Cd(1, 2 or 5 μM) for the 3 h and stained with MDC(50 μM). Fluorescence particles in the cytoplasm indicate autophagic vacuoles. Microphotographs were shown as representative results from three independent experiments.

Changes in expression of the autophagy marker protein LC3-II were examined to determine whether treatment of cells with Cd results in induction of autophagy in OBs. As shown in Fig. 2A,B, Cd (2μM) increased lipidated LC3-II levels in treated OBs, and levels decreased in a time-dependent manner. LC3-II expression was upregulated in cells exposed to Cd (1 and 2 μM) compared to the control (Fig. 2E). Interestingly, this concentration range (1–5 μM) was essential for Cd to induce apoptosis in OBs. In addition, alterations in beclin 1 and Atg5, which is required for formation of autophagosomes, were investigated in Cd-treated OBs. Cd treatment (1 and 2 μM) led to an increase in the expression level of beclin 1 and Atg5 as compared to the control, and a decrease in a time dependent manner in OBs (Fig. 2A,C,D,F–H). These data suggested that low level of Cd treatment might cause an accumulation of autophagosomes, with the level of autophagy decreasing in a time dependent manner.

Cd-induced autophagic flux was determined by MDC staining. Control cells exhibited diffuse and weak fluorescence (Fig. 2I). However, the OBs treated with Cd for 1 h exhibited an increase in the number of MDC-labeled vesicles. These data further demonstrate that Cd can induce autophagosome formation in OBs.

### Autophagy inhibits Cd-induced apoptosis in Obs

**Figure 3:**
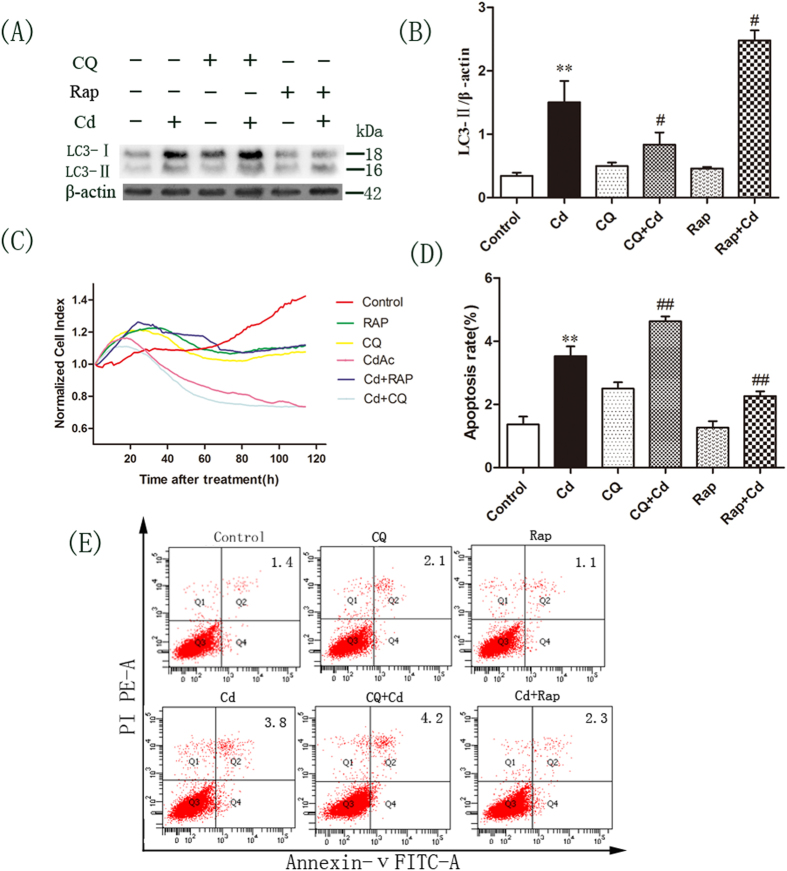
Autophagy regulated Cd-induced apoptosis in OBs. OBs were treated with Cd (2 μM) alone, or pretreated with RAP (100 nM) for 1 h or with CQ (5 μM) for 30 min followed by Cd (2 μM) for a further 3 h. (**A**) Western blot analyses of LC3 expression in treated cells. Blots for LC3-II (**B**) in OBs were semi-quantified using Image LabTM software. (**C**) The population of apoptotic cell was calculated. Normalized cell index represents cell viability determined by RTCA in OBs after treatment. (**E**) Apoptosis was determined by flow cytometry for Annexin-V-FITC and propidium iodide (PI) dual labeling. Cells in the Q2 and Q4 quadrants represent apoptotic cells. The mean is present in the Q2 quadrant. (**D**) We calculated the population of apoptotic cell. Data are expressed as mean ± SD (n = 3) relative to control.^**^P < 0.01 in comparison to the control by one-way ANOVA. ^#^*P* < 0.05 in comparison to the Cd treatment by one-way ANOVA. ^##^*P* < 0.01 in comparison to the Cd treatment by one-way ANOVA.

The effect of autophagy on Cd-induced cell death in OBs was determined by evaluating the effect of RAP and CQ on the expression of LC3-II. CQ and RAP increased the expression of LC3-II compared with 2 μM Cd only (Fig. 3A). Flow cytometry with Annexin V and propidium iodide staining indicated that CQ activated Cd-induced apoptosis and increased the apoptosis rate from 3.8% to 4.2%. In contrast, RAP rescued Cd-induced apoptosis and decreased the apoptosis rate from 3.8% to 2.3% (Fig. 3D,E). An xCELLigence real-time cell analysis (RTCA) assay was performed by pre-treating cells with CQ or RAP prior to Cd treatment. As shown in Fig. 3C, Cd treatment caused a decrease in cell viability. Interestingly, pre-treatment of cells with CQ decreased cell viability compared to cells treated with Cd only. However, pre-treatment of cells with RAP partially reversed the cytotoxic effects of Cd.

### Inhibition of autophagy by beclin 1 siRNA increases the cytotoxic sensitivity of cells to Cd

**Figure 4:**
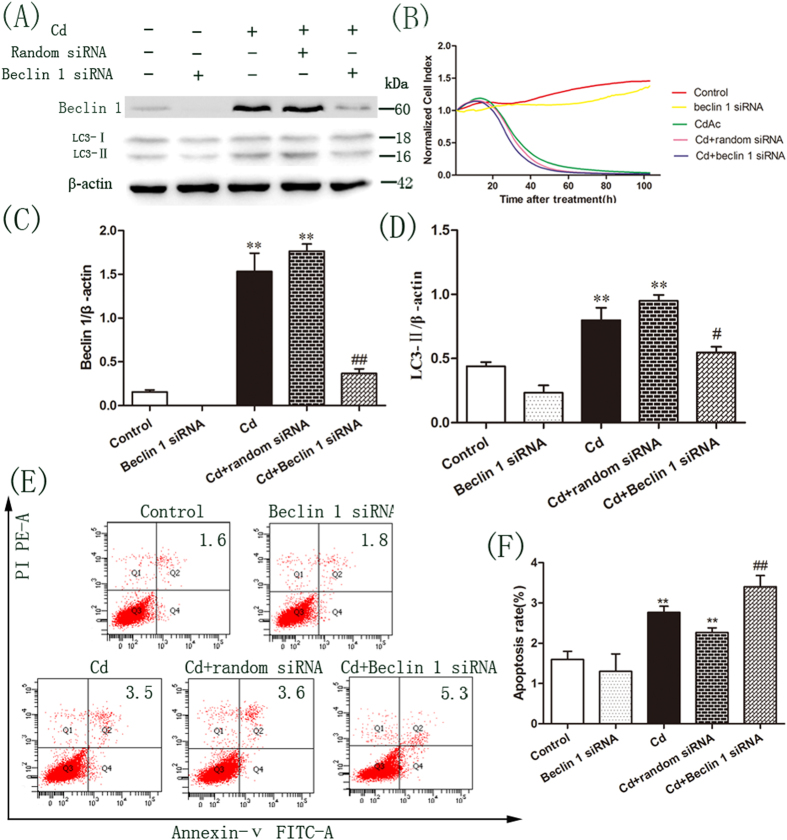
Beclin 1 siRNA inhibits autophagosome formation and enhances the cytotoxicity induced by Cd in OBs. OBs were treated with 2 μM Cd for an additional 3 h after transfection with random siRNA or beclin 1 siRNA for 24 h. (**A**) Immunoblotting for beclin 1 and LC3 using lysates from treated cells. Blots for LC3-II (**C**) and Beclin 1 (**D**) in OBs were semi-quantified using Image LabTM software. (**B**) Normalized cell index representing cell viability was determined by RTCA in OBs after treatment. (**E**) Apoptosis was determined by flow cytometry with Annexin V/PI staining. (**F**) We calculated the population of apoptotic cell. Data are expressed as mean ± SD (n = 3) relative to control.^**^P < 0.01 in comparison to the control by one-way ANOVA. ^#^*P* < 0.05 in comparison to the Cd treatment by one-way ANOVA. ^##^*P* < 0.01 in comparison to the Cd treatment by one-way ANOVA.

The role of autophagy in Cd-mediated cytotoxicity was further studied by knocking down beclin 1 expression using siRNA. Beclin1 plays an important role in autophagosome formation and is an essential component of the class III phosphatidy-linositol 3-kinase complex^29^. The expression of beclin 1 was markedly suppressed in OBs transfected with beclin 1 small interfering RNA (siRNA) compared to random siRNA (Fig. 4A,C). Accordingly, cells transfected with beclin 1 siRNA showed a reduced level of LC3-II accumulation after Cd treatment when compared with the random siRNA control (Fig. 4A,D). Consistent with the results using CQ, the cytotoxic effect of Cd was significantly increased by blocking beclin 1 expression (Fig. 4B). Similarly, the degree of apoptosis induced by Cd was also enhanced when beclin 1 expression was reduced by the specific siRNA (Fig. 4E,F).

## Discussion

Since the health hazards associated with Cd exposure were first reported in the 1940s, the toxic effect of Cd has been well characterized^30^,^31^,^32^,^33^. Due to its diverse toxic effects, extremely protracted biological half-life (approximately 20–30 years in humans) and worldwide anthropogenic mobilization, it is classified as one of 126 priority pollutants by the US Environmental Protection Agency^34^. Previous studies on Cd have focused on chronic low Cd exposure, mainly from dietary sources, which damages organ function and human health, rather than acute, lethal exposure^35^. Among these reports, studies have emerged that demonstrate the toxic influence of chronic low Cd exposure on bone mineral density^36^,^37^,^38^,^39^. One potential molecular mechanism of Cd-induced bone toxicity is osteoblast apoptosis, which is responsible for bone remodeling^40^.

Autophagy, which is morphologically characterized by the appearance of ‘double-membrane’ vacuoles, is a catabolic process that disposes of various cytoplasmic components, including protein aggregates and organelles. It is dependent upon a large group of Atgs, which are conserved between yeast and humans^41^. LC3 is the mammalian homologue of the yeast protein Atg8. It is recognized as a specific biochemical marker for autophagy. Some LC3-I is converted into LC3-II by autophagy induction. Subsequently, LC3-II is tightly bound to autophagosomal membranes. The autofluorescent agent MDC was introduced as a specific autophagolysosome marker to analyze the autophagic process^42^. MDC staining can be used to detect autophagic vacuoles (AVs). When cells were observed with a confocal fluorescence microscope, AVs stained by MDC appeared as distinct dot-like structures distributed within the cytoplasm or localizing in the perinuclear regions. Despite the fact that the molecular mechanisms of Cd-induced apoptosis are well understood^14^,^43^,^44^, few studies have investigated autophagy induced by Cd^45^. There is some evidence to suggest that that autophagy and apoptosis are functionally related^46^,^47^. However, autophagy may serve a positive function in reducing Cd-induced apoptosis^48^,^49^.

In the present study, both autophagy and apoptosis are induced in OBs during the course of Cd treatment. The results demonstrate that the autophagic form of LC3-II was increased after Cd treatment in OBs. This study also provides evidence that Cd was able to upregulate other important autophagosome-regulatory genes such as beclin 1 and Atg5. Interestingly, the expression of LC3- II and beclin 1 decreased in a time-dependent manner. Overall, these results indicate that apoptosis induced by Cd was accompanied by autophagy in OBs.

In the current study, the contribution of autophagy to the survival of OBs during Cd-induced apoptosis was investigated using the autophagy inhibitor CQ and autophagy inducer RAP. CQ-mediated autophagy inhibition increases the levels of LC3-II proteins, enhances Cd-induced cell apoptosis and increases cytotoxicity in OBs. Conversely, RAP activated Cd-induced autophagy, inhibited Cd-induced cell apoptosis and alleviated OB cytotoxicity. To further clarify the role of autophagy in Cd-induced cell death, beclin1 siRNA was used to knock down beclin 1 and evaluated the role of autophagy more directly. Consistent with the results using CQ, transfection of beclin 1 siRNA effectively inhibited autophagosome formation and enhanced the cytotoxicity induced by Cd in OBs. These findings suggest that Cd-induced autophagy might provide a self-defense mechanism for OBs and activation of autophagy may enhance the therapeutic efficacy of Cd in the treatment of osteoporosis.

In conclusion, this study demonstrates that autophagy is induced in OBs along with Cd-induced apoptosis. Inhibition of autophagy can result in potentiation of the proapoptotic effect of Cd, whilst activation of autophagy can rescue the apoptosis induced by Cd. These findings suggest that appropriate modulation of autophagy could provide a new strategy for Cd-induced osteoporosis therapy.

## Materials and Methods

### Reagents

Cadmium acetate (CdAc_2_), chloroquine (CQ), rapamycin (RAP), dansylcadaverine (MDC), anti-LC3 (Lot#: 065M4757V) and Hoechst 33258 were purchased from Sigma-Aldrich (St. Louis, MO, USA). The Annexin V-FITC apoptosis detection kit was purchased from BD Biosciences (San Diego, CA, USA). Dulbecco’s modified Eagle’s medium (DMEM) and fetal bovine serum (FBS) were obtained from Gibco (Grand Island, NY, USA). Trypsin was obtained from Amresco (Solon, OH, USA). Antibodies against Bax (Ref. No. : 03/2013), Bcl-2 (Ref. No. : 01/2013), cleaved-PARP (Ref. No. : 12/2012), autophagy gene 5 (Atg5) (Ref. No. : 01/2013), β-actin (Ref. No.: 06/2012) and horseradish peroxidase (HRP)-conjugated goat anti-rabbit immunoglobulin G (IgG) (Ref. No. : 10/2012) were purchased from Cell Signaling Technology (Boston, MA, USA). Anti-beclin 1 (Lot#: J0112), were purchased from Santa Cruz Biotechnology (Santa Cruz, CA, USA). Enhanced chemiluminescence (ECL) solution was obtained from Thermo Fisher Scientific (Waltham, MA, USA). Other chemicals and reagents were purchased locally and were all at analytical grade.

### Cell isolation and cell culture

Sprague–Dawley rats used in this study were purchased from the Laboratory Animal Center at Yangzhou University (Yangzhou, China). This study was approved by the Animal Care and Use Committee of Yangzhou University and was carried out in accordance with the Guide for the Care and Use of Laboratory Animals by the National Research Council.

Cranial OBs were obtained from 18–19 day-old Sprague–Dawley rat fetuses. The calvarias were incubated with 0.25% (w/v) trypsin at 37 °C for 10 min, then cut into slices and incubated with 0.1% collagenase at 37 °C for 40 min. The isolated primary OBs were cultured with DMEM supplemented with 10% FBS, 2 mM L-glutamine, 100 U/ml penicillin and 100 μg/ml streptomycin at 37 °C in a humidified atmosphere of 5% CO_2_ for 10–12 d. The phenotype was determined using an alkaline phosphatase (ALP) staining kit according to the manufacturer’s protocol (Beyotime Institute of Biotechnology, Jiangsu, China) (S1 Fig.).

### Cell viability measurement

Cell activity was monitored using the RTCA system (Roche, Mannheim, Germany). The background level was first determined by loading 100 μl/well of culture medium (DMEM with 10% FBS) into a 16-well E-plate. Changes in OB activity was monitored by seeding approximately 10,000 cells/well in the E-plate and culturing for 14 h in order for the cells to adhere and reach their proliferative phase. The impedance was measured every minute for the first hour and then every 15 min from 2–14 h.

Cells were treated with Cd (2 μM) alone, or pretreated with RAP (100 nM) for 1 h followed by Cd (2 μM), or pretreated with CQ (5 μM) for 30 min followed by Cd (2 μM). Following treatment, the impedance was measured every 15 min. The normalized cell index relative to a specified reference time point was determined by RTCA software.

### Hoechst 33258 staining

Cells (30 × 10^4^ cells/mL) were seeded on coverslips in six-well flat-bottomed plates. Following treatment with Cd (1, 2, 5 μM) for 3 h, cells were fixed with 4% paraformaldehyde at room temperature for 30 min, washed twice with PBS and permeabilized with 0.1% Triton X-100 in 0.1% sodium citrate. The cells were stained with Hoechst 33258 for 5 min at room temperature and then washed three times with PBS. Apoptosis was evaluated by imaging under a fluorescence microscope (Leica 2500; Leica Corporation, Germany).

### Western blot analysis

Total proteins (60–80 μg) were separated on 10–15% SDS-polyacrylamide gels and transferred to nitrocellulose membranes. The membranes were incubated with tris-buffered saline containing 0.05% Tween 20 and 5% nonfat milk to block nonspecific binding. The membranes were incubated overnight at 4 °C with antibodies against Bcl-2, Bax, cleaved-PARP, beclin 1, LC3 or β-actin (1:1000 dilution), followed by incubation with the corresponding HRP-conjugated secondary antibodies (1:5000 dilution). Protein detection was performed by enhanced chemiluminescence. All assays were performed in triplicate.

### MDC staining of autophagic vacuoles

Cells (30 × 10^4^ cells/mL) were seeded on coverslips in six-well flat-bottomed plates. Following treatment with Cd (1, 2, 5 μM) for 3 h, cells were fixed with 4% paraformaldehyde at room temperature for 30 min, washed twice with PBS and permeabilized with 0.1% Triton X-100 in 0.1% sodium citrate. Autophagic vacuoles were labeled with 0.05 mmol/L MDC in PBS at 37 °C for 10 min. The cells were then washed three times with PBS. Autophagic vacuoles in osteoblasts were observed under a confocal fluorescence microscope (Leica TCS SP8; Leica Corporation, Germany). The fluorescence intensity of MDC was measured at an excitation wavelength of 380 nm and emission wavelength of 530 nm.

### RNA interference of beclin 1

OBs were seeded in 6-well plates and incubated overnight. A control random siRNA or beclin 1-targeted siRNA (Invitrogen; 100 nmol/L) was transfected using an Amaxa Nucleofector (Primary Fibroblasts Basic Nucleofector Kit, VPI-1002, program T-041; Lonza) according to the manufacturer’s protocol. 24 h after transfection, cells were treated with Cd for an additional 3 h. Treated cells were collected and cell lysates were subjected to immunoblotting of beclin 1 and LC3. Cells were also processed for cell viability and apoptosis analysis.

### Apoptosis detection by flow cytometry

OBs were plated in six-well plates and incubated. OBs were treated with Cd (2 μM) alone, or pretreated with an autophagy inducer (100 nM rapamycin [RAP]) for 1 h followed by Cd (2 μM) for a further 3 h, or pretreated with an autophagy inhibitor (5 μM chloroquine [CQ]) for 30 min followed by Cd (2 μM) for a further 3 h. After treatment, cells were harvested by trypsinization and washed twice in PBS. After staining with the combination of Annexin-V/FITC Apoptosis Detection Kit, the cells were immediately analyzed by flow cytometer (FACS Calibur; Becton Dickinson, Franklin Lakes, NJ, USA).

### Statistical analysis

All experiments were performed independently a minimum of three times. The mean ± SD was determined by one-way ANOVA using SPSS software. The results were considered significant at *P < 0.05* and highly significant at *P < 0.01*.

## Additional Information

**How to cite this article**: Liu, W. *et al.* Role of autophagy in cadmium-induced apoptosis of primary rat osteoblasts. *Sci. Rep.* **6**, 20404; doi: 10.1038/srep20404 (2016).

## Supplementary Material

Supplementary Information
