# Supplementary material for: Role of autophagy in cadmium-induced apoptosis of primary rat osteoblasts
Source: Sci Rep. 2016 Feb 8;6:20404. doi: 10.1038/srep20404 (PMC4745071; doi:10.1038/srep20404)
Supplement: Supplementary Information [file srep20404-s1.pdf]

Role of autophagy in cadmium-induced apoptosis of primary rat osteoblasts

Wei Liu, , Nannan Dai , Yi Wang, Chao Xu, Hongyan Zhao, Pengpeng Xia, Jianhong Gu, Xuezhong Liu, Jianchun Bian, Yan Yuan , Jiaqiao Zhu* and Zongping Liu*

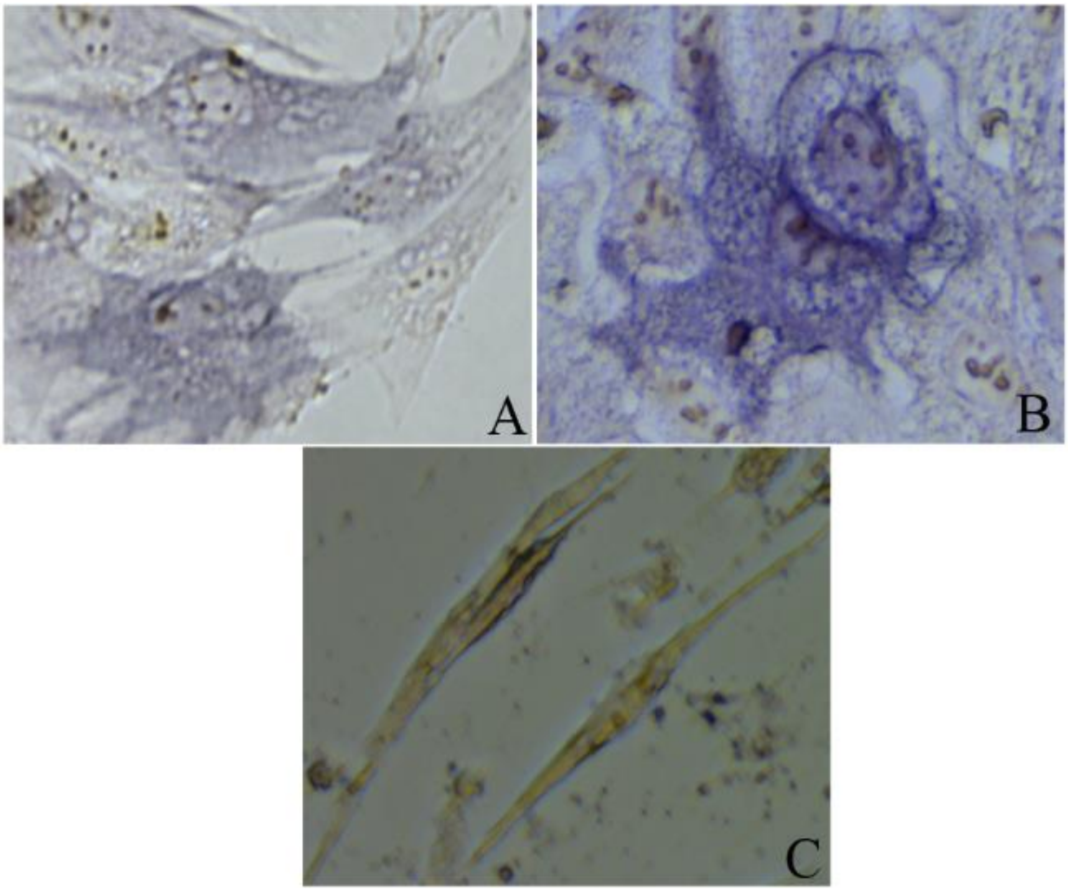

S1 Fig. The homogeneity test of OB population by alkaline phosphatase (ALP) staining. (A) OBs were cultured for 72 h to reach the typical period in which OB secrete alkaline phosphatase. The ALP of OBs was stained using the BCIP/NBT assay. ALP staining positive OBs showed visible blue granules distributed in the cytoplasm; the cell nuclei were negative. (B) The staining of ALP with UMR-106 showed deeper blue staining. The intensity of the color depends on the activity of ALP. (C) ALP staining of chicken embryo fibroblast (CEF) was negative.
